# How changes in depression severity and borderline personality disorder intensity are linked – a cohort study of depressed patients with and without borderline personality disorder

**DOI:** 10.1186/s40479-024-00247-2

**Published:** 2024-02-19

**Authors:** John J. Söderholm, J. Lumikukka Socada, Jesper Ekelund, Erkki Isometsä

**Affiliations:** 1 Department of Psychiatry, University of Helsinki and Helsinki University Hospital, Helsinki, Finland; 2 Department of Psychiatry, University of Helsinki, P.O. Box 22, Helsinki, FI-00014 Finland

**Keywords:** Borderline personality disorder, Major depressive disorder, Bipolar disorder, Borderline personality disorder severity index, Major depressive episode, Depression, Borderline, Personality disorder, Cohort study

## Abstract

**Background:**

Borderline personality disorder (BPD) is often complicated by comorbid major depressive episodes (MDEs), which can occur as part of major depressive disorder (MDD) or bipolar disorder (BD). Such comorbidity is related to worse outcomes in both disorders. Subsyndromal features of BPD are also common in depression. However, studies of simultaneous changes in BPD and depression severities are scarce, and their interactions are poorly understood.

**Aims:**

Studying the associations between changes in BPD and depression symptoms over the course of an MDE.

**Methods:**

In a 6-month naturalistic cohort study of MDE/BPD, MDE/MDD, and MDE/BD patients (*N* = 95), we measured change in BPD features between baseline and six months with the Borderline Personality Disorder Severity Index (BPDSI), an interviewer-rated instrument quantifying recent temporal frequency of BPD symptoms. We examined changes in BPD severity and their correlation with depression severity and other clinical measures and compared these across patient groups.

**Results:**

There were significant reductions in BPD severity, both in number of positive BPD criteria (-0.35, sd 1.38, *p* = 0.01672) and in BPDSI scores (-4.23, SD 6.74, *p* < 0.001), reflecting mainly a reduction in temporal frequency of symptoms. These were similar in all diagnostic groups. In multivariate regression models, changes in depression severity independently associated with changes in symptoms in the BDSI. This relationship was strongest in MDE/BPD patients but was not found in MDD patients without BPD.

**Conclusions:**

In the six-month follow-up, BPD features in MDE patients alleviated mainly by decreasing temporal symptom frequency and intensity. In BPD patients with comorbid MDE, changes in both conditions are strongly correlated.

## Introduction

Major depressive episodes (MDEs) can occur as part of major depressive disorder (MDD) or bipolar disorder (BD) [1]. Borderline personality disorder (BPD) is associated with a significantly increased risk of these mood disorders, with lifetime prevalence rates of MDD around 70% and BD around 20% [2–4]. Conversely, in MDE patients, comorbid BPD is common, with rates around 10% in MDD and 20% in BD [5]. Comorbid BPD in depression is correlated with a less favourable prognosis, increased risk of relapse, and increased risk of suicide attempts, affecting treatment [6–10]. Hence, the course of comorbid BPD is relevant for the prognosis and treatment of depression patients, and vice versa.

### The reciprocal relationship between BPD and mood disorders

According to several long-term cohort studies, BPD is not a static condition, instead the symptoms of BPD tend to ameliorate over time, with the great majority of patients reaching symptomatic remission in long-term follow-up, although functional impairments seem more persistent [11–15].The prevalence of depression in BPD also tends to decline over time but remains relatively high in follow-up, and relapses are common [16].Over long-term follow-up of patients diagnosed with both mood disorders and BPD, there is evidence of bidirectional negative effects on outcome in MDD/BPD but less robustly in BD/BPD [7]. A previous prospective cohort of MDD patients found a significant correlation between decline in depression severity and number of positive personality disorder (including but not limited to BPD) criteria and self-reported neuroticism [17, 18]. The factors underlying these relationships are likely to be complex. For instance, since a diagnosis of BPD is usually based on information obtained in a diagnostic interview, it can, in a depressed patient, be influenced by such factors as autobiographical, attentional, and emotional cognitive biases related to depression [19], with BPD symptoms seeming more pronounced during an MDE and less severe during remission. The DSM-5 recognizes this issue and explicitly warns against misdiagnosis of BPD in these circumstances: ”Because the cross-sectional presentation of borderline personality disorder can be mimicked by an episode of depressive or bipolar disorder, the clinician should avoid giving an additional diagnosis of borderline personality disorder based only on cross-sectional presentation without having documented that the pattern of behaviour had an early onset and a long-standing course” [1]. Still, PD diagnoses made during an MDE seem to have important prognostic implications, and a BPD diagnosis can be made also during an acute MDE, ascertaining that BPD symptoms have been present also when the patient is not acutely depressed [20]. How the symptomatology of BPD changes over the course of an MDE is not well known, however, and more detailed study of this issue would deepen our understanding of how these commonly comorbid disorders influence each other.

In longitudinal follow-up, BPD exhibits both trait-like (i.e. temporally stable) and state-like (more dynamic) features, with the stable component, or *BPD proneness*, closely correlated with Five Factor Model traits (i.e. descriptive normative personality traits), such as neuroticism, previously linked to BPD [21]. Examining how BPD feature severity changes over time and whether this change correlates with changes in depression severity in different patient groups (such as depression patients with and without BPD) would illuminate these issues further.

### Categorical and dimensional aspects of BPD

There is long-standing discussion on whether personality disorders are best described using categorical or dimensional diagnoses [22, 23]. BPD is still conceptualized as a categorical diagnosis in the main DSM-5 model, but the DSM also includes an alternative, hybrid approach that takes both traits and level of functioning into account [24], and ICD-11 utilizes a primarily dimensional approach based on functioning, with trait-based descriptors (including *borderline pattern*) being optional [1, 25]. Thus, attempts have been made to reconcile categorical diagnosis with more theoretically, and perhaps prognostically, valid dimensional evaluation.

One approach to quantifying BPD severity is according to the number of positive DSM-5 diagnostic criteria or otherwise measured symptoms, with more symptoms signifying higher severity [12, 15]. However, since the rating concerns long-standing patterns apparent from (at least) young adulthood, these are by design not very sensitive to change over the short or even medium-term (weeks to months), and quick changes in these might reflect a change in recall and other cognitive biases rather than personality change. More accurate methods are also available; the Borderline Personality Disorder Severity Index (BPDSI) is an interviewer-rated, valid, and reliable instrument for quantifying recent BPD symptom frequency (mostly, in 8 of 9 symptom domains by rating how often symptoms occur) in greater detail [26], and has been used as a measure of treatment efficacy in trials of psychotherapeutic, pharmacological, and neuromodulatory treatment of both BPD and persistent depressive disorders [27–30]. Consistent with the view of BPD as a dimensionally occurring phenomenon that may increase the risk of mood disorders, subsyndromal symptoms of BPD are more common in depression than in the general population. For example, the non-BPD participants in this study had a significantly higher BPDSI score at baseline than previously found in healthy controls [31, 32]. Nonetheless, to the best of our knowledge, there are no studies comparing the changes in dimensionally measured BPD feature severity in depression patients with MDD or BD, and with and without BPD, over time. A diagnosis of BPD, according to the DSM-5, is made based on established (retrospective) symptom patterns of high pathology, pervasiveness, and persistence over adult lifetime. Therefore, one might reasonably assume that prospectively assessed BPD symptom frequency and severity (measured, for instance, with the BPDSI) may be more temporally stable in BPD than non-BPD patients; however, this has not been previously investigated using methods precisely quantifying symptom frequency and severity.

### Aims of the study

We evaluated the changes in BPD feature severities over the course of an MDE in MDD and BD patients, including patients with and without comorbid BPD. We hypothesized, firstly, that frequency and intensity of BPD symptoms, measured by the BPDSI and BPD criteria, would ameliorate over the course of the MDE, correlating with attenuation of depression severity. Secondly, BPD symptoms were hypothesized to be more stable in BPD patients than in others. If a correlation between the changes in BPD symptom and depression severities emerged, we intended to explore whether such a relationship was also present for anxiety and BPD symptoms.

## Method

This naturalistic cohort study with a follow-up of at least 6 months is based on the Bipolar – Borderline Depression (BiBoDep) cohort.

### Recruitment and sampling

Our recruitment process has been described in more detail elsewhere [31, 33]. We recruited patients with depression starting outpatient treatment at one of two psychiatric care clinics of the City of Helsinki, Finland, with a total catchment area of 234 000 adults.

We aimed to include adequate numbers of MDE patients with MDD, BD, and/or comorbid BPD, applying stratified randomized sampling to achieve this. Based on information in the referrals, we divided all incoming depression referrals (*n* = 1655) into six preliminary strata by (i) sex and (ii) probable diagnosis: (a) MDD, (b) MDE in BD, (c) MDE with comorbid BPD. We prioritized patients in strata that were underrepresented in our sample at that time. If there were multiple possible recruits within the preferred stratum, recruitment order was determined randomly with a random number generator available online at random.org. Patients were contacted by phone, and those providing preliminary consent were met and given additional oral and written information about the study.

Consenting patients were then interviewed with the Structured Clinical Interview for DSM-IV, i.e. SCID-I and SCID-II [34, 35]. The diagnostic interviews were thorough, lasting around three hours per patient at baseline, and the diagnostic evaluation was also based on information in patients’ clinical charts. Diagnostic reliability, assessed with independent rating of videos of these interviews, was found to be excellent, with a Cohen’s kappa of 1.00 for MDD, 0.90 for BD, and 0.89 for BPD. We examined current depression severity with the Montgomery Åsberg Depression Rating Scale (MADRS) [36].

### Inclusion and exclusion criteria

Inclusion criteria were a current MDE, a MADRS score of 15 or more, and age of 18–50 years. Exclusion criteria have been described in more detail previously [31, 33], but included psychotic illness or ongoing psychotic symptoms, active substance use disorders, antisocial personality disorder, lacking proficiency in the Finnish language, and significant neurocognitive or sensory impairments.

### Sample and subcohort assignment

Altogether 124 patients were included in the study at baseline. Our patients were divided into three subcohorts, such that all patients with MDD without BPD belonged to one subcohort (MDD, *n* = 50), patients with BD belonged to the second subcohort (MDE/BD, *n* = 43), and patients with comorbid BPD belonged to the third subcohort (MDE/BPD, *n* = 31). BD patients with comorbid BPD were assigned to the BD subcohort if they had type I BD, otherwise we assigned them to either the MDE/BD or the MDE/BPD subcohort depending on main clinical picture at and preceding baseline. Unclear cases were discussed in the study group, and a consensus decision of subcohort assignment was reached.

### Baseline evaluation

In addition to the diagnostic interviews and MADRS, we also asked study participants to complete the Beck Depression Inventory II (BDI II) [37] and the Overall Anxiety Severity and Impairment Scale (OASIS) [38].

### Borderline personality disorder severity index

We evaluated severity of recent BPD symptom severity with the BPDSI [26]. The BPDSI rates 70 items, comprising occurrence frequency (for 8/9 symptoms) and severity (the identity disturbance symptom) of instances of the 9 DSM-IV (and 5) symptoms during the preceding 3-month period, yielding a total sum score measuring overall BPD severity, as well as symptom level subscores. In rating the BPDSI, we also had access to patients’ clinical charts, with information regarding possible suicide attempts and other relevant information. The BPDSI interviews lasted around 1 h per patient.

### Follow-up

We had a follow-up period of at least 6 months, after which we met with patients again, repeating the SCID, MADRS, and BPDSI. Altogether 95 patients were available for follow-up. Remission from the MDE was achieved by 56.8% of patients, with no significant differences between cohorts: MDE/MDD 56.4%, MDE/BD 60.6%, and MDE/BPD 52.2%, *p* = 0.8196 [10]. In assessing clinical course (relevant for e.g. the BPDSI and SCID), we had access to clinical charts as well as a biweekly online follow-up questionnaire consisting of an expanded version of the Personal Health Questionnaire-9 [10]. Of note, the focus of both SCID-II interviews was whether PD criteria were currently met, based on information regarding participants’ lifetimes. As previously reported, we found no significant differences between drop-outs and non-drop-outs in subcohort or clinical data [10].

### Analyses

Data were assembled into a database using SQLite, version 3.35.5 (SQLite Team, www.sqlite.org) and analysed with R version 4.2.2 (R Foundation, www.r-project.com) on PC computers running Microsoft Windows 11. We used parametric and non-parametric tests as appropriate, analysis of variance testing, and linear regression models. Significance testing of changes over time was done with paired samples t-tests comparing baseline and later scores, and change magnitude between groups was examined with ANOVA comparisons of the change in scores.

## Results

Demographic and clinical characteristics of the cohort and the subcohorts at baseline and follow-up are reported in Table 1.

**Table 1:** Demographic and clinical characteristics at baseline and follow-up

Sample		MDD (*n* = 39)		MDE/BD (*n* = 33)		MDE/BPD (*n* = 23)		Total sample (*n* = 95)		
		mean / n	sd / %	mean / n	sd / %	mean / n	sd / %	mean / n	sd / %	p
Age		31.41	10.29	32.79	9.62	27.98	7.10	31.06	9.46	0.167
Sex	Female	21	53.8%	25	75.8%	16	69.6%	62	65.3%	0.168
	Male	18	46.2%	8	24.2%	7	30.4%	33	34.7%	
BPD		0	0.0%	2	6.1%	23	100.0%	25	26.3%	< 0.001
Bipolar		0	0.0%	33	100.0%	6	26.1%	39	41.1%	< 0.001
	Type I	0	0.0%	8	24.2%	0	0.0%	8	8.4%	
	Type II	0	0.0%	25	75.8%	6	26.1%	31	33.7%	
MDE features	Melancholic	12	30.8%	12	36.4%	10	43.5%	34	35.8%	0.585
	Atypical	9	23.1%	3	9.1%	6	26.1%	18	18.9%	0.196
**Baseline**	MADRS	23.62	5.98	22.21	7.05	21.52	6.35	22.62	6.45	0.426
	BDI II	27.45	9.91	30.44	11.78	32.94	9.54	29.88	10.63	0.215
	OASIS	11.69	3.33	12.85	3.73	12.22	5.08	12.22	3.94	0.467
	BPDSI	14.81	7.33	16.65	6.75	25.60	8.42	17.48	8.29	< 0.001
**Follow-up**	MADRS	15.43	9.71	12.96	12.00	16.50	4.77	14.79	9.86	0.455
	BDI II	19.32	12.11	19.04	16.58	26.27	10.85	20.54	13.72	0.201
	OASIS	9.16	4.48	8.44	5.33	10.40	4.42	9.15	4.76	0.499
	BPDSI	11.61	8.33	11.88	7.13	19.03	7.85	13.17	8.28	0.005
**Correlations**	MADRS	0.48		0.32		0.21		0.36		
	BDI II	0.45		0.68		0.39		0.53		
	OASIS	0.44		0.52		0.55		0.47		
	BPDSI	0.66		0.48		0.63		0.69		

*Notes* Correlations are between baseline and follow-up scores. MDD = Major depressive disorder, MDE = Major depressive episode, BD = Bipolar disorder, BPD = Borderline personality disorder, MADRS = Montgomery Åsberg Depression rating scale, BDI II = Beck depression inventory II, OASIS = Overall anxiety severity and impairment scale, BPDSI = BPD severity index. p refers to Anova or Fisher’s exact Χ^2^ testing of intra-subcohort differences

### Changes in categorical BPD diagnoses

For the vast majority of patients, there was no change in their BPD diagnostic status at follow-up (i.e. most BPD diagnoses were still valid, and most patients not meeting BPD criteria at baseline did not meet them at follow-up either). Only two patients diagnosed with BPD at baseline did not meet diagnostic criteria at follow-up, whereas two other patients who had not met BPD criteria at baseline now did so; thus, there was no change in the net sum of BPD patients. The patients no longer meeting full BPD diagnostic criteria at follow-up were MDD patients sorted into the BPD subcohort, who had met 5 (the diagnostic minimum) of the BPD diagnostic criteria at baseline and 3 and 4, respectively, at follow-up, one of them had achieved remission from MDE as well. Patients with new BPD diagnoses were BD subcohort patients with type II BD, meeting 3 and 4 BPD criteria at baseline and 3 more (i.e. 6 and 7) at follow-up, respectively, one of them having achieved remission from MDE.

### Changes in number of positive BPD criteria

The mean number of positive BPD criteria was 3.12 (sd 2.34) at baseline and 2.77 (sd 2.42) at follow-up in the whole cohort, signifying a mean change of 0.35 (sd 1.38), which was statistically significant (*p* = 0.017); the effect size was small (Hedge’s g = 0.14). Subcohortwise, the criteria sums were 1.64 (sd 1.56) and 1.15 (sd 1.39) in MDD, 2.61 (sd 1.34) and 2.45 (sd 1.80) in MDE/BD, and 6.35 (sd 1.15) and 5.96 (sd 1.22) in MDE/BPD subcohorts, respectively, with no significant differences between the cohorts in the amount of change (*p* = 0.59). We did not find evidence of significant differences in magnitudes of change between diagnostic groups (BPD vs. non-BPD, BD vs. non-BD).

### BPDSI total and subscores

Changes in BPDSI total and subscores are reported in Table 2. The effect size for total BPDSI change was moderate (Hedge’s g = 0.5).

**Table 2:** Changes in BPDSI Total and Subscores from Baseline to Follow-up

Sample	MDD			MDE/BD			MDE/BPD			Whole Cohort			
	Mean	sd	p	Mean	sd	p	Mean	sd	p	Mean	sd	p	ANOVA p
Total	-3.20	6.52	0.005	-4.78	7.06	0.001	-5.73	6.77	0.005	-4.23	6.74	< 0.001	0.416
Sensitivity to Abandonment	-0.10	0.88	0.387	-0.20	1.23	0.223	0.10	1.25	1.000	-0.10	1.07	0.192	0.682
Unstable Relationships	0.13	0.77	0.392	0.14	0.80	0.546	-0.28	1.18	0.489	0.06	0.87	0.616	0.265
Identity Disturbance	-0.35	1.42	0.113	-0.66	1.72	0.026	-1.04	1.12	0.005	-0.59	1.49	< 0.001	0.312
Impulsivity	-0.05	0.43	0.493	-0.19	0.72	0.200	-0.24	0.67	0.221	-0.14	0.59	0.053	0.511
Suicidality and Self-Harm	-0.16	0.62	0.085	-0.08	0.61	0.073	-0.48	0.86	0.059	-0.19	0.67	0.004	0.174
Affective Hyperreactivity	-1.55	2.26	< 0.001	-1.76	1.91	< 0.001	-0.97	1.46	0.036	-1.52	2.01	< 0.001	0.470
Feelings of Emptiness	-0.62	2.79	0.242	-1.68	2.70	< 0.001	-1.63	3.04	0.064	-1.18	2.82	< 0.001	0.261
Difficulties in Anger Control	-0.36	1.09	0.126	-0.14	1.43	0.468	-0.46	1.47	0.263	-0.30	1.28	0.048	0.695
Dissociative and Paranoid Symptoms	-0.13	0.91	0.451	-0.21	1.16	0.345	-0.74	1.82	0.173	-0.27	1.21	0.070	0.246

*Notes* MDD = Major depressive disorder, MDE = Major depressive episode, BD = Bipolar disorder, BPD = Borderline personality disorder, sd = standard deviation. p refers to significance testing of amount of change (i.e. comparison of baseline to follow-up scores); ANOVA p refers to analysis of variance significance testing of differences between cohorts

Grouping patients into diagnostic groups, BPD patients (regardless of subcohort) had a significant mean total BPDSI score change of -4.91 (sd 7.32, *p* = 0.017) and bipolar patients − 5.18 (sd 7.54, *p* < 0.001). There were no significant differences in the amounts of change between BPD and non-BPD patients (*p* = 0.674) or between BD and non-BD patients (*p* = 0.328).

The mean change in BPDSI score was − 3.21 (sd 6.82) in patients who still fulfilled MDE criteria at follow-up, whereas those who were in a state of remission from MDE had a mean change of -5.02 (sd 6.66); the difference between remitted and non-remitted patients was non-significant (*p* = 0.2385).

### Correlates of change in BPDSI

**Fig. 1:**
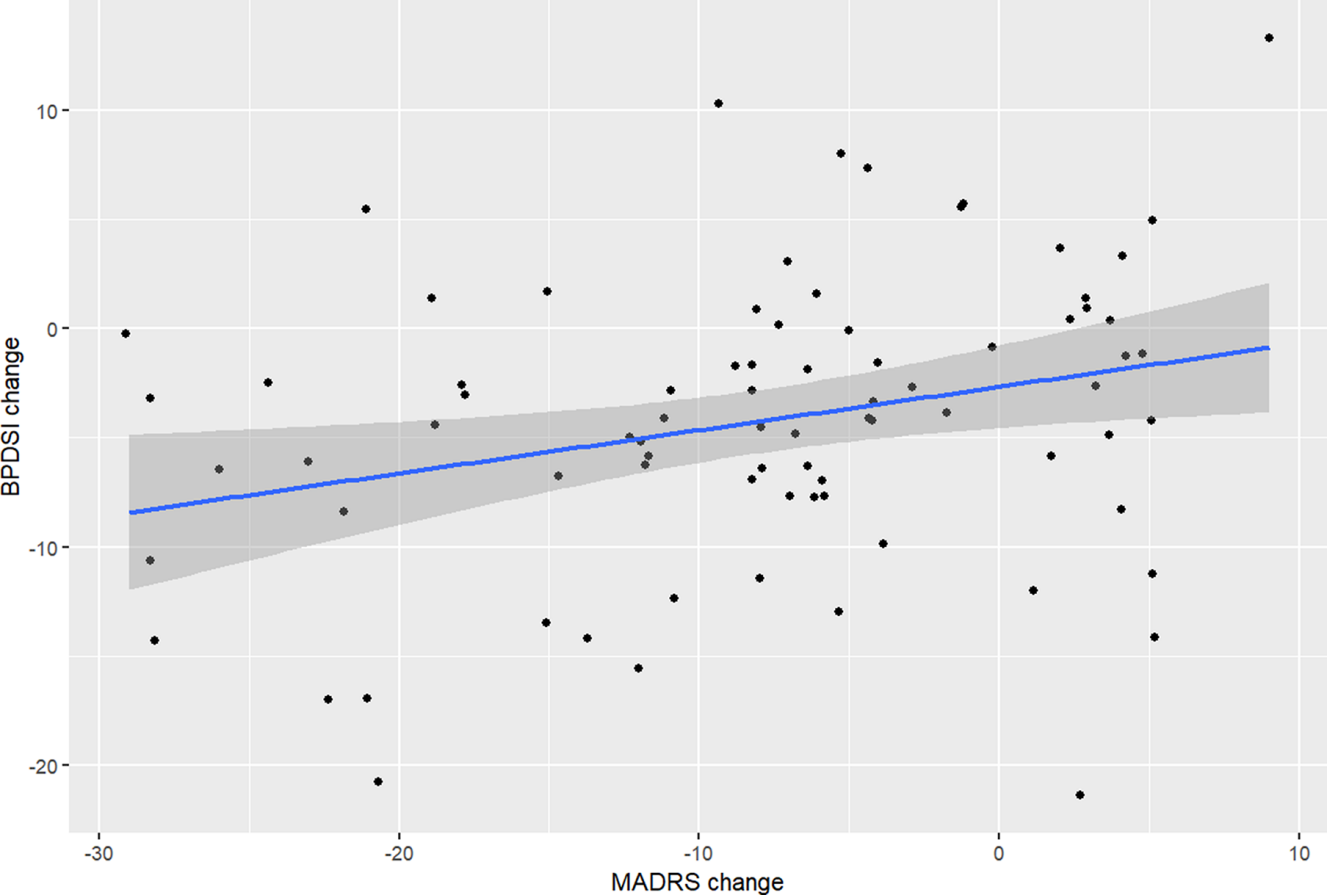
Correlation between changes in BPDSI and MADRS during study in whole cohort

**Fig. 2:**
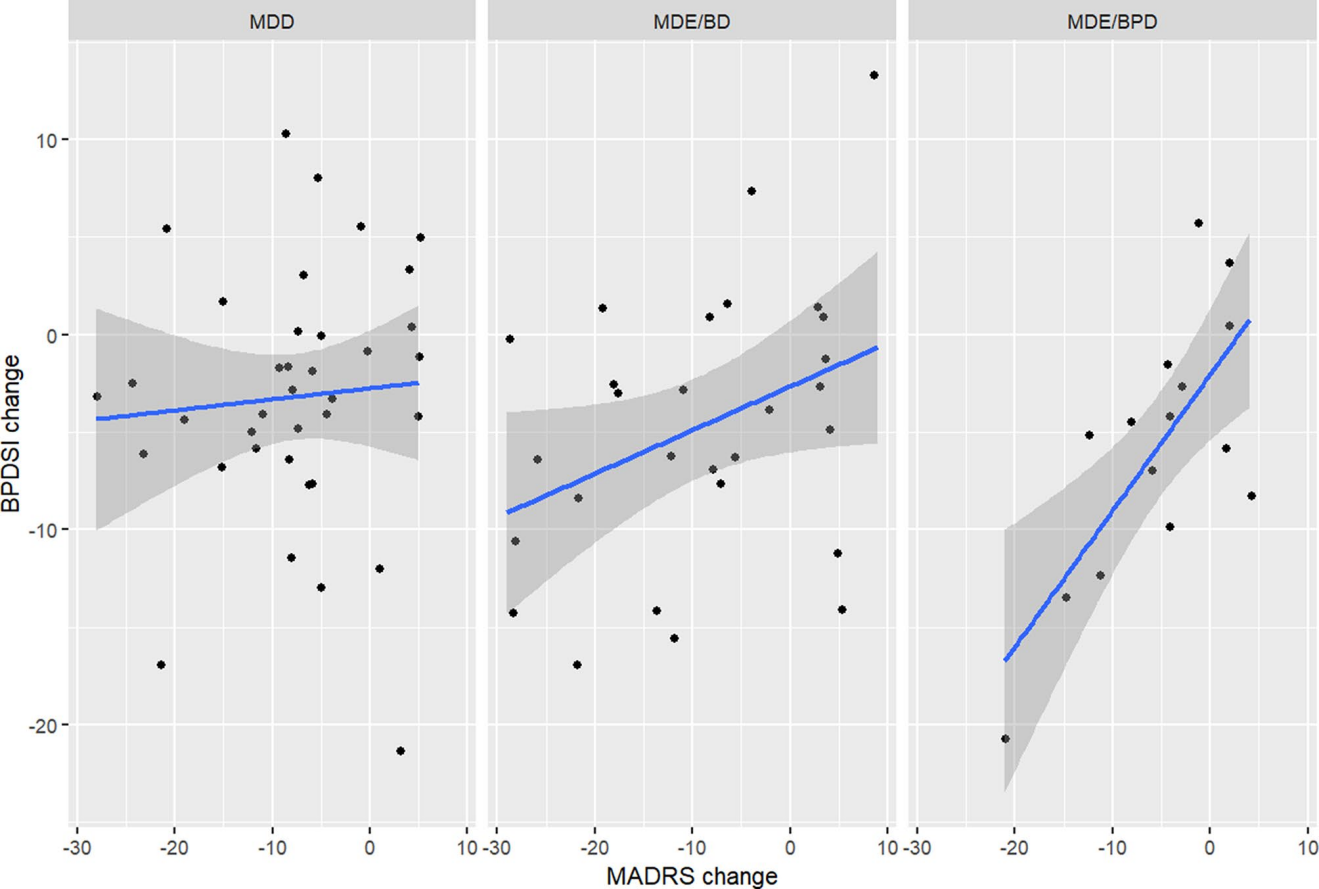
Correlation between changes in BPDSI and MADRS during study by subcohort

We examined the correlations between changes (from baseline to follow-up) in BPD feature severity (as measured by the BPDSI) and depression severity (as measured by the MADRS) in the whole sample and in the subcohorts graphically (see Figs. 1 and 2) and numerically; the correlation in the whole cohort was small but significant (*r* = 0.28, 95% cl 0.06–0.47, *p* = 0.01185). The correlation was strong and significant in the MDE/BPD subcohort (*r* = 0.73, 95% CI 0.38–0.90, *p* = 0.02) but non-significant in the MDE/BD (*r* = 0.37, 95% CI -0.006–0.65, *p* = 0.055) and MDD subcohorts (*r* = 0.07, 95% CI -0.25–0.39, *p* = 0.660). When analysed by diagnostic groups (i.e. all BPD or BD patients grouped together regardless of subcohort assignment), the correlations were significant for both groups: BPD patients (*r* = 0.67, 95% CI 0.27–0.87, *p* = 0.004) and BD patients (*r* = 0.42, 95% CI = 0.09–0.67, *p* = 0.016). There was also a significant correlation between changes in BPDSI and BDI-II (*r* = 0.31, 95% CI 0.06–0.53, *p* = 0.018). Changes in OASIS were not correlated with BPDSI changes (*r* = -0.047, 95% CI -0.266–0.178, *p* = 0.685).

We examined the robustness of the correlation between change in BPDSI and MADRS through linear regression models. When controlled by age, sex, change in OASIS score, and BPD and BD diagnostic status, the MADRS change remained a significant predictor of BPDSI change (*p* = 0.007), which the other variables were not; however, this model was not significant in itself (F 1.858 on 6 and 71 df, *p* = 0.1001). Stepwise dropping of non-significant variables yielded a significant (F 4.251 on 2 and 75 df, *p* = 0.01784) model in which MADRS change was significantly (estimate 0.240, SE 0.083, 95% CI 0.074–0.406, *p* = 0.005) correlated with BPDSI change when controlled by OASIS change (the correlation of the latter being non-significant: estimate − 0.269, SE 0.17711, 95% CI -0.622–0.084, *p* = 0.132).

## Discussion

### Main findings

In this 6-month cohort study of major depressive patients with and without borderline personality disorder (BPD), we found that BPD feature severity decreased significantly over time both in BPD patients and in patients with subsyndromal BPD features. This was noted both in reduced number of positive BPD criteria in repeated diagnostic interviews and in a lower BPD severity index (BPDSI) score, reflecting lower frequency and intensity and of borderline symptoms. Whereas the effect size for the change in number of positive BPD diagnostic criteria was small, the effect size for change in BPDSI scores was moderate, indicating that this instrument was more sensitive to changes in the occurrence frequencies of symptoms. There were no significant differences in the amelioration of BPD symptoms over time between unipolar and bipolar depression patients, nor between BPD and non-BPD-patients. Changes in BPD feature severity were significantly correlated with changes in depression severity. Interestingly, this correlation was significantly stronger in BPD patients than in others, and was non-significant in MDD patients without comorbid BPD. Put differently, even MDE patients without a BPD diagnosis had significant BPD features at baseline, which became less marked during follow-up; however, in contrast to BPD patients, there was no evidence of this amelioration being correlated to how much their depression symptoms diminished.

### BPD outcome after follow-up

No net change occurred in BPD point prevalence over time, supporting earlier reports that diagnostic change seems faster in mood disorders than in BPD [15]. Thus, our time frame might have been too short to detect such changes on a categorical diagnostic level. The changes in number of BPD criteria were also less marked than those in BPDSI scores. These findings thus might reflect the aim of the SCID-PD interview, which is primarily to evaluate the significance of symptoms over patients’ entire lifespans, rather than only recently. Our findings are not in conflict with the prevailing view of BPD as a partly dynamic disorder with a clear tendency toward symptomatic amelioration over time [14, 21], as there was a significant, although modest, reduction in BPD feature severity measured with BPDSI scores (corresponding to symptoms occurring less frequently or strongly) as well as with BPD criteria. As a concrete example of the magnitude of changes in this time period, at baseline the score for the affective hyperreactivity category in the MDE/BPD subcohort was approximately 7 (rounded from 7.3), signifying that the average patient had experienced these symptoms weekly, and after follow-up the mean score was around 6 (6.1), which corresponds to symptoms in this domain occurring twice every three weeks. This dynamic seems to be valid both for depressive patients meeting the full BPD criteria and for those with subsyndromal symptoms, in line with viewing BPD as a dimensionally occurring phenomenon, rather than a categorical entity.

### Correlations between changes in BPD and depression severity

Changes in depression and BPD severity were linked also when controlled for other relevant factors (such as anxiety and main diagnoses). However, when examining how changes in BPD symptom severity are correlated with changes in depression severity, we found marked differences between depressive patients with and without BPD; the correlation in BPD patients was significant and moderately high, but we found no evidence of a correlation in MDD patients without BPD. Considering the large difference between correlations (r 0.67 vs. 0.07), this seems unlikely to be simply an inferential (type II) error. This finding was contrary to our a priori hypothesis and warrants further study, but we wish to offer some possible explanations. Depression confers negative cognitive biases [19], and BPD patients might potentially be more affected by these biases than others, perhaps as a function of what has been described as BPD proneness or personality features, such as neuroticism [21], which would increase the correlation between the two. Interestingly, changes in anxiety (as measured by the OASIS) and BPDSI change did not correlate in any subgroup; attentional and cognitive biases in anxiety are more related to perceived external threats than to the self [39], and thus, changes in these may not influence the experience and occurrence of BPD symptoms as strongly (or, indeed, aet all). This difference in correlations may also reflect a difference in the unmeasured precipitants of depression. For example, the role of external triggers of symptomatic decline (such as adverse life events) might differ for BPD and non-BPD patients. In addition to potential differences in these triggers per se, BPD patients might, due to their affective hyperreactivity, have a tendency to react to these triggers more strongly, which would also explain differences in symptom change correlations. Another possibility is that (fullblown, syndromal) BPD is a cause of MDE, and that depression symptoms alleviate when BPD features alleviate in these patients, but not in others. Emotional dysregulation is closely linked to the BPD phenotype, and has been shown to mediate the effect of childhood maltreatment on risk of later depression [40], and decline in emotional dysregulation might thus explain both alleviation of depression and BPD symptoms. Alternatively, as the relationship of depression and borderline features may be reciprocal and bidirectional [7], this observed pattern might be conceptualized as an alleviation of a more global illness process rather than of two discrete disorders [41].

### BPD symptom subdomains

In addition to an overall alleviation of BPD symptoms, we found significant reduction in five (out of nine) of the DSM symptom subdomains: identity disturbance, suicidality/self-harm, identity disturbance, feelings of emptiness, and difficulties in anger control; all, but the last, were highly significant. A reduction in suicidality is to be expected, as depression (generally) lessened over time and has indeed been reported in this cohort (using other methods and measures) previously [9]. In one earlier cohort study of BPD symptomatic change, the results were somewhat different, as impulsivity was the first to change and affective symptoms the last, with interpersonal and cognitive symptoms lying between the two [15]. Another study found amelioration in impulsive, affective, and interpersonal symptoms, but not in cognitive symptoms, and a third reported approximately similar rates of decline in all of the DSM-5 symptom domains of BPD over 10 years [13]. Differences in time frames and instruments used and, perhaps most importantly, our focus being on MDE patients may contribute to the variability of results.

### Strengths and limitations

Strengths of this study include the clinically and theoretically relevant comparative design of three central depressive groups of treatment-seeking psychiatric care patients, the prospective study design, and the use of valid and reliable dimensional measures of BPD symptomatology and other symptom severity. The study also has some limitations. The follow-up time of 6 months was chosen in order to examine change over the course of an MDE but precludes drawing longer term conclusions. Since the research interviews were done by the same researcher for each patient, they were not blinded to diagnoses when assessing, e.g. the BPDSI. Although inter-rater reliability was excellent for main diagnoses, it was not assessed for all measures, including the MADRS and the BPDSI. Our sample size was moderate, but even so, we made significant new findings. Since we investigated outpatient psychiatric care patients, confirmation of our results in other settings is required. We focused on MDD patients, and the relationships between BPD and depression severities might conceivably be different in persons with minor depression or subsyndromal depression symptoms. Although we found interesting and suggestive relationships, the study design precludes drawing firm conclusions about causal relationships – for instance, we did not assess the possible role of psychosocial stressors as triggers for MDE, and thus, any changes in these,or other common causes of both BPD and MDD, such as emotional dysregulation, over the follow-up-period could explain changes in both depression and BPD severity. Alternatively, some features of BPD and depression may overlap at least indirectly or otherwise influence each other (for instance depressive dysphoria increasing the risk of anger and/or self-harm, and BPD-linked interpersonal problems might worsen depressive symptoms); the precise mechanisms of such reciprocal effects were largely beyond the scope of this study. The BPDSI instrument mostly focuses on symptom frequency, which was detectable; however, other mechanisms by which BPD feature severity may decrease, not identified using these methods, are also possible. What we see is thus dependent on what is being sought. Still, we would argue that the BPDSI is a methodological improvement over less detailed methods used in earlier research, such as number of positive BPD criteria in the SCID-PD, and quite specific for the DSM symptoms of BPD. Use of other dimensional assessment models of personality pathology, such as the DSM-5 alternative model and the ICD-11 are likely to illuminate these issues further, and could be combined with BPDSI or other measures for detecting changes in symptoms in future research.

## Conclusions

In conclusion, we found interesting similarities, but also some differences, between changes in BPD severities over the course of an MDE in patients with MDD, BD, and/or BPD. The view of BPD as a partially dynamic phenomenon with both trait- and state-like components is refined by a deepened understanding of the relationship of frequently co-occurring BPD and depression. Specifically, the frequency and severity of BPD symptoms tend to ameliorate when recovering from depression, and one way in which this change takes place is through a lessening in frequency of both observable and subjective symptoms of BPD. Change in BPD and depression symptom severities seem to correlate in BPD patients, but not in non-BPD patients; this phenomenon warrants replication and further investigation. Seeing change in BPD is partly dependent on using instruments (such as the BPDSI) calibrated to detect change over the relevant period.

## Data Availability

Due to European (GPDR) and Finnish national data privacy legislation, and lack of participant consent for data sharing, we are unable to provide patient-level data. Group-level data are available from the authors upon reasonable request.

## Abbreviations

BD: Bipolar Disorder
BDI II: Beck Depression Inventory II (BDI II)
BPD: Borderline Personality Disorder
BPDSI: Borderline Personality Disorder Severity Index
DSM-5: Diagnostic and Statistical Manual, 5th edition
ICD-11: International Classification of Disease, 11th edition
MADRS: Montgomery Åsberg Depression Rating Scale
MDE: Major Depressive Episode
MDD: Major Depressive Disorder
OASIS: Overall Anxiety Severity and Impairment Scale
SCID: Structured Clinical Interview for DSM
SCID-PD: SCID Personality Disorder Section
